# Supplementary material for: Stroke in adults with primary intracranial tumours
Source: J Neurol. 2025 Feb 17;272(3):212. doi: 10.1007/s00415-025-12929-0 (PMC11832572; doi:10.1007/s00415-025-12929-0)
Supplement: Supplementary file 1 — Supplementary file1 (DOCX 743 KB) [file 415_2025_12929_MOESM1_ESM.docx]

**SUPPLEMENTARY MATERIAL**

**METHODS**

*Statistical analysis*

The initial Cox proportional hazards models for survival after hospital discharge and stroke recurrence are given below. The results are shown as Forest plots in the Supplemental Figures.

*Initial Cox proportional hazard model for survival in patients discharged from hospital*

Expected hazard of death at time t = (Expected hazard of death at time 0) · exp(β_1_(Stroke) + β_2_(Age) + β_3_(Pre-stroke mRS score) + β_4_(mRS score at hospital discharge) + β_5_(Malignancy) + β_6_(Radiotherapy) + β_7_(Surgery) + β_8_(Chemotherapy) + β_9_(Sex) + β_10_(Stroke consultant visit within 24 hours of stroke)

where: time 0 is time of tumour diagnosis; Stroke was haemorrhagic or ischaemic; Malignancy was malignant or benign; Sex was Male or Female; and radiotherapy, surgery, chemotherapy and Stroke consultant visit within 24 hours were binary Yes/No variables (Fig. S1B). Sex and Stroke Consultant visit within 24 hours of stroke were removed from the final model.

*Initial Cox proportional hazard model for stroke recurrence*

Expected hazard of stroke recurrence at time t = (Expected hazard of stroke recurrence at time 0) · exp(β_1_(Smoking) + β_2_(Antiplatelet/anticoagulation initiation post-stroke) + β_3_(Statin initiation post-stroke) + β_4_(Antihypertensive initiation post-stroke) + β_5_(Radiotherapy) + β_6_(Chemotherapy) + β_7_(Conventional stroke aetiology) + β_8_(Index stroke type) + β_9_(Age) + β_10_(Stroke consultant visit within 24 hours of stroke) + β_11_(Tumour type) +β_12_(Surgery)

where time 0 is the time of the index stroke; conventional stroke aetiology is any one of the TOAST diagnoses or hypertension; index stroke type is ischaemic or symptomatic intracranial haemorrhage; tumour type was benign or malignant; and Stroke consultant visit within 24 hours and surgery were binary Yes/No variables (Fig. S2A). Index stroke type, Age, Stroke consultant visit within 24 hours, tumour type and surgery were removed in the final model.

*Expression for relative risk of an ischaemic stroke within six months of the diagnosis of a benign primary intracranial tumour*

For a study population, Let

B = number of patients with benign intracranial tumour in the population

M = number of patients with malignant intracranial tumours in the population

I_M_ = number of patients having an index ischaemic stroke within 6 months of the diagnosis of their malignant primary intracranial tumour

I_B_ = number of patients having an index ischaemic stroke within 6 months of the diagnosis of their benign primary intracranial tumour

RR_IM_ is the relative risk of a patient having an index ischaemic stroke within 6 months of the diagnosis of their malignant primary intracranial tumour with respect to the general population

RR_IB_ is the relative risk of a patient having an index ischaemic stroke within 6 months of the diagnosis of their benign primary intracranial tumour compared with the general population

R_AIS_ is the risk of ischaemic stroke over 6 months in the general population.

Then

The relative prevalence of benign to malignant tumours in a population, Prev(B/M) = B/M

The relative risk of a patient having an index ischaemic stroke within 6 months of the diagnosis of their malignant primary intracranial tumour with respect to the general population,

RR_IM_ = (I_M_/M)/ R_AIS_ = I_M_ / (M x R_AIS_)

Rearranging,

R_AIS_ = I_M_ / (M x RR_IM_)

The relative risk of a patient having an index ischaemic stroke within 6 months of the diagnosis of their benign primary intracranial tumour compared with the general population,

RR_IB_ = (I_B_/B)/ R_AIS_ = I_B_/(B x R_AIS_)

Substituting for R_AIS_

RR_IB_ = I_B_/(B x I_M_ / (M x RR_IM_))

RR_IB_ = (I_B_ x RR_IM_)/((B/M) x I_M_) = (I_B_ / Prev(B/M)) x ((RR_IM_) / I_M_)

*R Citations*

R version 4.3.0 (2023-04-21 ucrt)

Platform: x86_64-w64-mingw32/x64 (64-bit)

Running under: Windows 11 x64 (build 22621)

Matrix products: default

*R Packages*

1. Wickham H, Bryan J (2023). _readxl: Read Excel Files_. R package version 1.4.2, <https://CRAN.R-project.org/package=readxl>.
2. <duncan@wald.ucdavis.edu>. GDTcCpiscf'bDTL (2021). _excel.link: Convenient Data Exchange with Microsoft Excel_. R package version 0.9.10, <https://CRAN.R-project.org/package=excel.link>.
3. H. Wickham. ggplot2: Elegant Graphics for Data Analysis. Springer-Verlag New York, 2016. Wickham H, Vaughan D, Girlich M (2023). _tidyr: Tidy Messy Data_. R package version 1.3.0, <https://CRAN.R-project.org/package=tidyr>.
4. R. Pruim, D. T. Kaplan and N. J. Horton. The mosaic Package: Helping Students to 'Think with Data' Using R (2017). The R Journal, 9(1):77-102.
5. Yihui Xie (2023). knitr: A General-Purpose Package for Dynamic Report Generation in R. R package version 1.42.
6. Yihui Xie (2015) Dynamic Documents with R and knitr. 2nd edition. Chapman and Hall/CRC. ISBN 978-1498716963
7. Yihui Xie (2014) knitr: A Comprehensive Tool for Reproducible Research in R. In Victoria Stodden, Friedrich
8. Leisch and Roger D. Peng, editors, Implementing Reproducible Computational Research. Chapman and Hall/CRC. ISBN 978-1466561595
9. Bache S, Wickham H (2022). _magrittr: A Forward-Pipe Operator for R_. R package version 2.0.3, <https://CRAN.R-project.org/package=magrittr>.
10. Wickham H, Hester J, Bryan J (2023). _readr: Read Rectangular Text Data_. R package version 2.1.4, <https://CRAN.R-project.org/package=readr>.
11. Waring E, Quinn M, McNamara A, Arino de la Rubia E, Zhu H, Ellis S (2022). _skimr: Compact and Flexible Summaries of Data_. R package version 2.1.5, <https://CRAN.R-project.org/package=skimr>.
12. Iannone R, Cheng J, Schloerke B, Hughes E, Lauer A, Seo J (2023). _gt: Easily Create Presentation-Ready Display Tables_. R package version 0.9.0, <https://CRAN.R-project.org/package=gt>.
13. Kassambara A, Kosinski M, Biecek P (2021). _survminer: Drawing Survival Curves using 'ggplot2'_. R package version 0.4.9, <https://CRAN.R-project.org/package=survminer>.
14. Therneau T (2023). _A Package for Survival Analysis in R_. R package version 3.5-5, <https://CRAN.R-project.org/package=survival>.
15. Terry M. Therneau, Patricia M. Grambsch (2000). _Modeling Survival Data: Extending the Cox Model_. Springer, New York. ISBN 0-387-98784-3.

**Supplementary Tables**

**Table S1. Factors Associated with 30-Day Stroke Mortality in Primary Intracranial Tumour Patients**

| **Variables** | **Univariate Analysis** | | **Multivariate Analysis** | |
| --- | --- | --- | --- | --- |
|  | **Unadjusted OR** | **p-value** | **Adjusted OR** | **p-value^1^** |
| Age (increase per year) | 1.04 *(1.02-1.06)* | *8x10^-4*^* | 1.03 *(1.00 – 1.06)* | *0.03** |
| Pre-Stroke mRS (per unit increase in mRS score)  (for every 1-unit increase) | 1.59 *(1.29-1.98)* | *2x10^-5^** | 1.47 *(1.16-1.88)* | *0.001** |
| Tumour (malignant / benign) | 0.75 *(0.32-1.63)* | *0.49* | 1.12 *(0.43-2.80)* | *0.81* |
| Stroke (ischaemic / haemorrhagic) | 0.91 *(0.45-1.84)* | *0.79* | 0.53 *(0.22-1.21)* | *0.14* |
| *¹ p-value calculated using logistic regression modelling* | | | | |
| ** denotes statistically significant results* | | | | |
| *Numbers in parentheses are 95% confidence intervals.*  *mRS = modified Rankin Scale* | | | | |
| *mRS = modified Rankin Scale* | | | | |

*All tumours were malignant or benign; all strokes were ischaemic or haemorrhagic*

**Table S2. Factors Associated with Stroke Recurrence in patients with Primary Intracranial Tumours**

| **Variables** | **Univariate Analysis** | | **Multivariate Analysis** | |
| --- | --- | --- | --- | --- |
|  | **Unadjusted OR** | **p-value^1^** | **Adjusted OR** | **p-value^1^** |
| Tumour type (malignant / benign) | *1.36 (0.74-2.45)* | *0.32* | *0.77 (0.18-2.85)* | *0.71* |
| Tumour – Ischaemic Stroke colocalisation  (for every 1-unit increase) | *1.02 (0.43-2.30)* | *0.96* | *0.67 (0.20-1.96)* | *0.48* |
| Conventional Stroke Causes (TOAST or Hypertension) | *0.94 (0.47-1.80)* | *0.85* | *1.12 (0.43-2.80)* | *0.81* |
| Radiotherapy before index stroke | *3.45 (1.39-8.54)* | *7x10^-3^** | *8.80 (2.02-47.09)* | *6x10^-3^** |
| Radiotherapy after index stroke | *2.03 (0.84-4.67)* | *0.10* | *3.22 (0.48-20.12)* | *0.21* |
| Smoking status – Ex-smoker | *1.50 (0.67-3.23)* | *0.31* | *1.44 (0.50-4.03)* | *0.49* |
| Smoking status – Current smoker | *1.58 (0.66-3.62)* | *0.29* | *1.73 (0.48-5.70)* | *0.38* |
| *Odds ratios are yes/no for explanatory variables unless stated* | | | | |
| *Numbers in parentheses are 95% confidence intervals.* | | | | |
| *¹ p-value calculated using logistic regression modelling*  *mRS = modified Rankin Scale* | | | | |
| ** denotes statistically significant results* | | | | |
| *TOAST = Trial of ORG 10172 in Acute Stroke Treatment classification system* | | | | |

**Table S3. Ischaemic Stroke Investigations**

| **Investigations** | **n** | **(%)** | |
| --- | --- | --- | --- |
| ECG | 120 | (86) | |
| Echocardiogram | 72 | (52) | |
| Lipid Profile | 62 | (45) | |
| HbA1c | 59 | (42) | |
| CT Angiogram | 53 | (38) | |
| Carotid Doppler | 53 | (38) | |
| 24-hour ECG | 47 | (34) | |
| **Total** | **139** | |  |

Investigations performed on 141 patients for ischaemic stroke and mixed strokes. An ECG had to be uploaded to electronic patient records for us to record it. However, a new patient electronic record system was introduced during the study period. ECGs performed prior to the introduction of the new system were not uploaded retrospectively on a routine basis.

**Supplementary Figures**

**
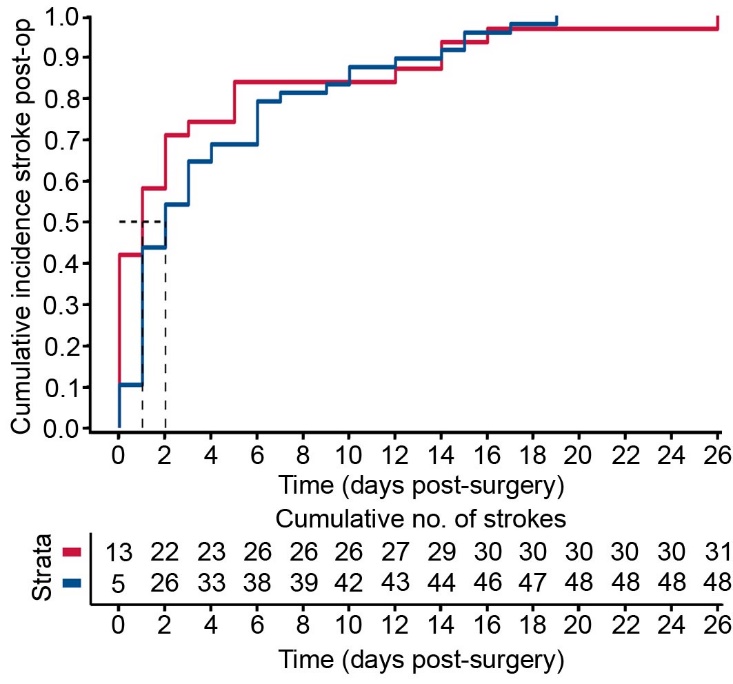
**

**Fig. S1 Index strokes after brain tumour surgery**

Cumulative incidence of ischaemic (blue) and haemorrhagic (red) index strokes after brain tumour surgery. Dashed lines denote the median time after surgery of ischaemic (2 days) and haemorrhagic (1 day) strokes. The plots do not include three patients who had mixed postoperative strokes.


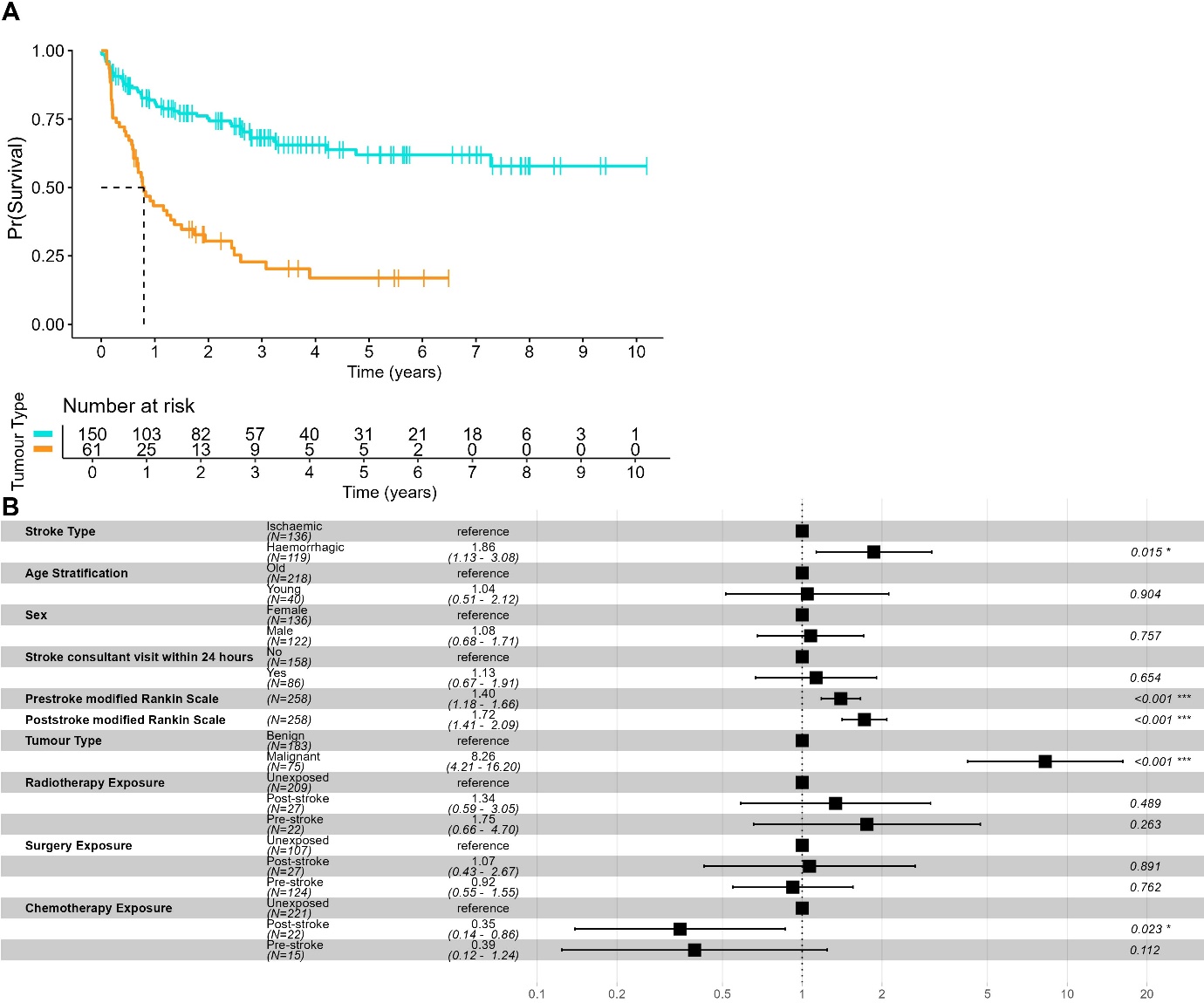


**Fig. S2.** **Survival of patients discharged from hospital following stroke**

(**A**) Kaplan-Meier plot of survival following stroke in patients who were discharged from hospital. (**B**) Forest plot for the initial statistical model for survival after hospital discharge following stroke. Sex and stroke physician review were removed from the final model.


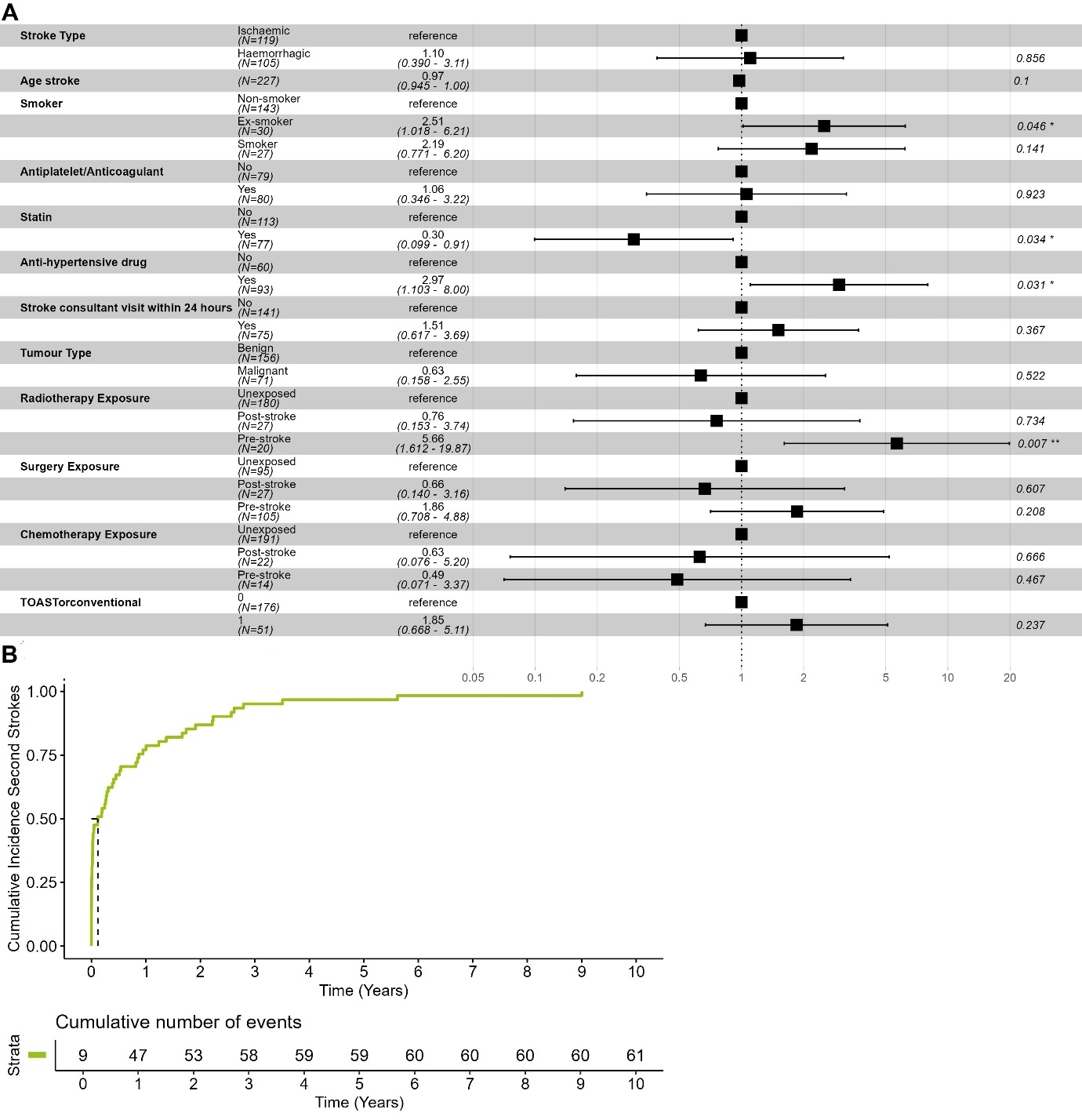


**Figure S3.** **Stroke recurrence**

(**A**) Forest plot of initial statistical model for stroke recurrence including asymptomatic ischaemic infarction. The explanatory variables removed from the final model were: stroke type; age at the time of first stroke; review by a stroke physician within 24 hours; tumour malignancy and surgery. (**B**) Cumulative incidence of clinically-symptomatic recurrent strokes.
